# The effects of text direction of different text lengths on Chinese reading

**DOI:** 10.1038/s41598-023-35859-1

**Published:** 2023-05-29

**Authors:** Yanqun Huang, Yifan Dong, Zhaojun Jiang, Peng Zhang, Jutao Li, Junyu Yang

**Affiliations:** 1 Key Laboratory of Mechanism Theory and Equipment Design of Ministry of Education, Tianjin University, Tianjin, 300354 China; 2 Tianjin Ren’ai College, Tianjin, 301636 China; 3 School of Automobile NCO, University of Army Military Transportation, Bengbu, 233011 Anhui China

**Keywords:** Human behaviour, Information technology

## Abstract

This study investigates the effects of text direction (horizontal and vertical) and length (long and short) on Chinese reading performance. The experiment enrolled 68 university students aged 19–29 years who were asked to read articles. We recorded reading times and measured recall after reading using a memory test and measured task load using the NASA-TLX scale. The results show that horizontal text was read faster than vertical text. When reading long texts, horizontal reading has a better memory effect than vertical reading. When reading short texts, the effect of text direction on memory was not significant. Moreover, the mental, physical, and temporal demands of horizontal text were lower than those of vertical text. These findings contribute to a better understanding of the impact of text direction, provide valuable suggestions for Chinese typography, and help readers obtain better reading outcomes.

## Introduction

Chinese is one of the most spoken languages in the world (counting only first-language speakers), with over 1.325 billion people whose mother tongue is Chinese^1^. There are many discrepancies between Chinese and alphabetic writing systems, such as that of English, because they represent different spelling system types. Chinese contains approximately 2500 commonly used characters, shaped as squares or rectangles^2^, with most words consisting of 1–4 characters, each comprising 1–36 strokes^3,4^. Therefore, Chinese characters contain more visual information than English letters. In addition, Chinese characters are equidistant and lack clear word boundaries^5^, and readers must segment the text into meaningful words through experience and conventions. This indicates the relative importance of cognitive and linguistic factors in Chinese reading^6^.

Text arrangement is part of the layout design, guiding the line of sight when reading, which has a significant impact on the reading experience^7^. For thousands of years, the traditional Chinese writing layout has followed a vertical arrangement (i.e., the word order is from top to bottom, and the line order is from right to left), reflecting long-standing Chinese culture and expressing an Oriental manner of cognition^8^. Today, vertical writing is primarily used for artistic and aesthetic purposes (such as short logos and covers), in scholarly works on long Chinese classical literature, and in cases where space is limited (such as spine and diagram representations). Chinese calligraphy is written vertically in both Simplified and Traditional Chinese forms. With an increase in cultural exchange between the East and West near the beginning of the twentieth century, horizontal typesetting began to appear in Chinese and developed in popularity. In 1956, the Chinese government actively promoted character reform and implemented simplified characters as well as a horizontal arrangement. Ever since then, books, newspapers, textbooks, and other reading materials have utilized horizontal layouts, coexisting with vertical long texts in classical books (Zhu, 2017) and vertical short texts in menus^9,10^, leading displays on TV^11^ and traffic signs^12^.

The Chinese language characteristics of square glyphs and equal word spacing enable flexible writing in different directions. Chinese can be read horizontally rightward and leftward, or vertically downward^13^. Previous research has shown that text orientation (i.e., the direction in which the text is read) affects reading performance and visual cognition^5,13–15^. Text length is also a key factor in user preference^16^ and reading accuracy and comprehension^17^. Nonetheless, no consensus has been reached on the superiority of horizontal or vertical reading orientation. Previous studies have largely been limited to analyzing speed and eye movements during reading, and it is unclear how text orientation affects memory and cognitive performance when reading texts of different lengths. Therefore, this study examines whether text orientation can improve the efficiency and cognitive ability of Chinese readers for different text lengths. The results of this study may serve as a reference for the layout and design of Chinese books and advertisements.

## Literature review

Text display formation affects both readability and reading performance. Numerous studies have investigated the impact of text typography on reading, including font style^18,19^, font size^20,21^, spatial layout^22^, and line/word spacing^5,23^. Text direction, in particular, is a vital factor in reading efficiency^13,24,25^. Byrne^25^ noted that the reading speed for rotated and marquee texts was lower than that for horizontal texts in the presentation of English. Similarly, Yu, Park^26^ and Porter and Arblaster^24^ reported that native English speakers read vertical texts significantly more slowly than horizontal texts because of their smaller visual spans when reading vertically. In contrast, owing to the characteristics of Chinese writing units, Chinese is suitable for flexible writing and reading in either the horizontal or vertical direction^13^.

Several studies have explored the effects of text orientation on Chinese reading. Early research by Miles and Shen^27^ used eye-tracking technology to study Chinese reading and found that the visual span was larger when reading vertical than horizontal material, and vertical reading seemed to have an advantage over horizontal reading in terms of reading speed. Further research by Chen and Carr^28^ with 27 Chinese college students (aged 20–26 years) yielded similar results, namely, that Chinese adults read vertical text faster and more accurately than horizontal text. However, subsequent studies reported different and even opposite conclusions. Sun, Morita^29^ found that the visual span of vertical reading was only 50% that of horizontal reading, and the fixation time was 10% longer. In a Chinese proofreading experiment, Chan and Ng^4^ obtained a similar finding; the proofreading speed for horizontal text was faster than that for vertical text, and text direction had a significant effect on proofreading preference, with participants rating horizontal text as better in terms of proofreading comfort, ease, and fatigue. Wang^5^ investigated different Chinese text layouts to determine whether they improved the reading speed and comprehension of Taiwanese children (aged 10 and 11), also concluding that horizontal text was read faster and comprehended better than vertical text. However, Yan and Pan^13^ argued that readers generated saccades more efficiently vertically than horizontally, suggesting that vertical reading is at least as efficient as horizontal reading for Taiwanese students (undergraduate and graduate) who have adapted to traditional Chinese characters. Researchers have explained that the inconsistency primarily lies in readers’ skills^29^. Interestingly, text direction may be more than just a convention, and different cultures may shape our visual cognition of certain types of text. Alternatively, certain textual directions may be selected to fit the biological characteristics of a particular population. Therefore, it is of great theoretical significance for future research to test the vertical and horizontal reading proficiencies of people from different language and cultural backgrounds.

Furthermore, text direction affects an individual’s cognitive processes, and a large number of eye movements may negatively influence readers’ searching and processing of information^30,31^. Chen and Lin^32^ found that text display type had a significant impact on cognitive load. Wang, Cui^14^ compared the visual recognition performance for horizontal and vertical layouts in both English and Chinese using word search and word understanding tasks, proposing that visual recognition performance in the horizontal layout was higher than that in the vertical layout; however, the differences were smaller in Chinese, owing to its greater layout flexibility. Ktori, Grainger^33^ investigated the effect of letter string display type (horizontal, vertical, and circular) on visual short-term memory and found that horizontally presented letter arrays had a selective advantage over vertical and circular arrays, which possibly used specialized coding mechanisms built upon years of reading experience.

This bias may be related to intrinsic differences in the horizontal and vertical extent of the observer’s visual system. The binocular visual field is a horizontally oriented ellipse, and vertical lines are generally closer to the boundary of the visual field than horizontal lines; thus, vertical lines usually appear longer^34^. Another explanation involves differences in directional eye movements, with vertical eye movements requiring more effort and taking longer than horizontal ones^35^. Horizontal eye movements increase interhemispheric brain activity, thus facilitating episodic memory retrieval^36,37^; they also increase recognition sensitivity and decrease response time in spatial memory tests compared to vertical eye movements^38^.

Meanwhile, text length has a major impact on reading performance. Long texts often result in low sentiment scores^16,39^. Jaeger and Ares^40^ suggest that performing a single highlighting task with a longer text is not as effective as splitting the task into two shorter texts. Conversely, in accelerated reading tasks, long texts can improve high-school students’ comprehension^17^. According to Andreassen and Bråten^41^, longer texts increase the need for a working memory system, whereas shorter ones are more dependent on decoding capacity^17^. Thus, how text length affects readers’ memory and cognition warrants further discussion.

In summary, there is no evidence that either vertical or horizontal reading has a biological advantage^42^. For readers with both horizontal and vertical reading skills, further research on the effects of text direction on other cognitive traits is required to determine which arrangement is advantageous in terms of attention, memory, and cognitive load. Therefore, to investigate the effects of text direction and text length on Chinese reading performance, we conducted an experiment using the professional online experimental psychology software program, PsychoPy^43^. We collected data on participants’ reading behavior, measured their recall using a memory test, and assessed their task load using the NASA-Task Load Index (NASA-TLX) scale. This study offers a fresh perspective on Chinese character arrangement and helps readers obtain better reading outcomes.

### Hypotheses

Based on previous research^35–38^, horizontal eye movements facilitate response and memory. Therefore, we hypothesize that users with both horizontal and vertical reading skills perform better when reading horizontal text.

H1: Horizontal or short text is faster to read than vertical or long text.

H2: Horizontal or short text facilitates better memory than vertical or long text.

H3: Horizontal or short text creates a lower task load than vertical or long text.

The independent variables were text direction and length, and the dependent variables were reading speed, memory, and task load.

## Methodology

This study was approved by the ethics committee of Tianjin University, and the experiments were performed in accordance with the approved guidelines.

### Experimental design

A 2 × 2 mixed factor design was adopted to study the effects of text direction and length on reading speed, memory, and task load. Text direction was the between-subjects factor with two levels: horizontal and vertical, and text length was the within-subjects factor with two levels, long and short. A mixed design was used to minimize participant fatigue and learning effects and to reduce the experimental error caused by individual differences among participants.

### Participants

Sixty-eight students at Tianjin University with different majors and grades participated in the experiment (33 men and 35 women), ranging in age from 19 to 29 years (M = 22.63, SD = 1.915). All participants were from mainland China and usually read books (long texts) horizontally and advertisements and posters (short texts) vertically, both of which are relatively common. All participants were computer users who had not previously participated in the relevant experiments. They had no known reading difficulties and had normal or corrected-to-normal vision. All participants signed an informed consent form prior to the experiment.

### Apparatus

The participants were seated in front of a 15.6-in. monitor with a resolution of 1280 × 1080 pixels for individual testing. The chair was adjustable according to individual conditions, and the participants’ eyes were approximately 60 cm away from the monitor. PsychoPy was used as an experimental tool to present the stimulus samples and record the operational responses. The experiment was performed in a brightly lit and quiet laboratory. Before the experiments, we minimized the possible influence of other variables such as screen brightness and network quality.

### Materials

Scientific and technological texts were used as experimental materials to reduce the influence of emotional factors on the participants^44^. The experimental materials consisted of one long and one short text. The long text was a popular science article excerpted from *A Brief History of Time*, with a total word count of 7766. The short text comprised several portions of science and technology news, approximately 300–400 words each in length, with a total word count of 4217 words. According to the readability formula, *y* = 0.8*x*_*1*_ + *x*_*2*_ (where *y* is the readability index, *x*_*1*_ is the average number of words per sentence, and *x*_*2*_ is the percentage of difficult words^45^), the readability levels of the two texts were 8.0 and 7.2, which were appropriate for the participants in this experiment^44^. The reading materials were provided in simplified Chinese (the participants’ native language). The participants had not read the articles prior to the experiment.

### Measurements

#### Reading speed

Reading speed is the number of words read per minute (wpm)^46^, and has been widely used to compare paper and digital reading^47^. The participants’ reading time was recorded to calculate reading speed. When the participants began reading, the PsychoPy program automatically recorded the reading time for each page to obtain the total time and calculated the reading speed according to the number of words in the article.

#### Memory

The participants' memory was measured using the Remember/Know (R/K) test^48^, a widely employed tool for gauging the nature and quality of memory^44,46^. The R/K test is based on two main types of retrieval responses: “remembering” and “knowing”, where remembering (R) was driven primarily by contextual-information and knowing (K) was driven mainly by information relevant to the items. This study measures the recall of episodic details, which is also known as episodic memory (R). For each article, there were ten questions that were sentences extracted from the original text, but with key words removed^44^. Four options were provided to the participants: the correct answer was a word omitted from the original text, and the other answers were similar to the correct answer but did not match the context. Memory was measured using the number of correct answers (out of 10 questions) and reaction time (average reaction time while answering the questions).

#### Task load

Task load was assessed using the widely accepted NASA-TLX scale^49^, developed by NASA’s Ames Research Center, a multidimensional instrument based on six dimensions: mental demand, physical demand, temporal demand, performance, effort, and frustration. Data shows that the scale is mostly applied in interface design or evaluation such as visual or auditory displays (31%)^50^. Participants were self-assessed according to their actual feelings while reading; the higher the participant’s score, the higher the task load.

### Experimental procedure

The participants were randomly divided into two groups of 34 each: a horizontal text group (aged 18–29 years, M = 22.59, SD = 1.971) and a vertical text group (aged 19–29 years, M = 22.68, SD = 1.887). Each participant was asked to read either a long or short text. The experiment used a Latin square design to balance reading order.

First, the experimenters recorded the participants’ personal information and verbally informed them of the experimental procedure and requirements. The participants were asked to read in a relaxed state and answer questions regarding the article. The participants were not informed of the content of the questions before the experiment, and no time limits or goals were set.

Next, the participants began the experimental program and read the experimental instructions, the English translation of which is as follows: “In the experiment, you need to read two texts and complete one content-related questionnaire after reading each text. Please read according to your personal reading habits and stay relaxed. Each text can be read only once. You can advance to the next page by clicking the space bar.” Subsequently, the participants pressed the space bar to reach a preparation page stating, “Please read the following text.”

Next, the participants began the formal reading process. The long text was displayed approximately evenly across 12 pages, and the short text was displayed across 10 pages, with 300 to 400 words per page. PsychoPy recorded browsing time on each page.

After fully reading the assigned text (either the long or short text), participants were required to complete a memory test form and task load scale. The memory test consisted of ten multiple-choice questions with each question comprising a sentence randomly selected from the original text, with a key word omitted. Four options, one key word, and three similar words were provided for each question. Participants were required to choose the response that they thought was in the original text. Subsequently, they completed the NASA-TLX scale to assess mental, physical, and temporal demand, and performance, effort, and frustration. After reading the first text, participants were given a 3-min break and then began reading the second text and completing the related questions.

The entire experiment took approximately 30 min.

The data and materials supporting the findings of this study are available from the corresponding author upon reasonable request.

## Results

Sixty-eight valid data points were collected. A two-factor repeated-measures analysis of variance (RM-ANOVA) was performed using SPSS software (IBM SPSS Statistics Subscription Trial, https://www.ibm.com/cn-zh/products/spss-statistics), with text length as the within-group variable and text direction the between-group variable, to determine significant differences in the effects of text direction and text length on reading speed, memory, and task load.

### Reading speed

**Figure 1:**
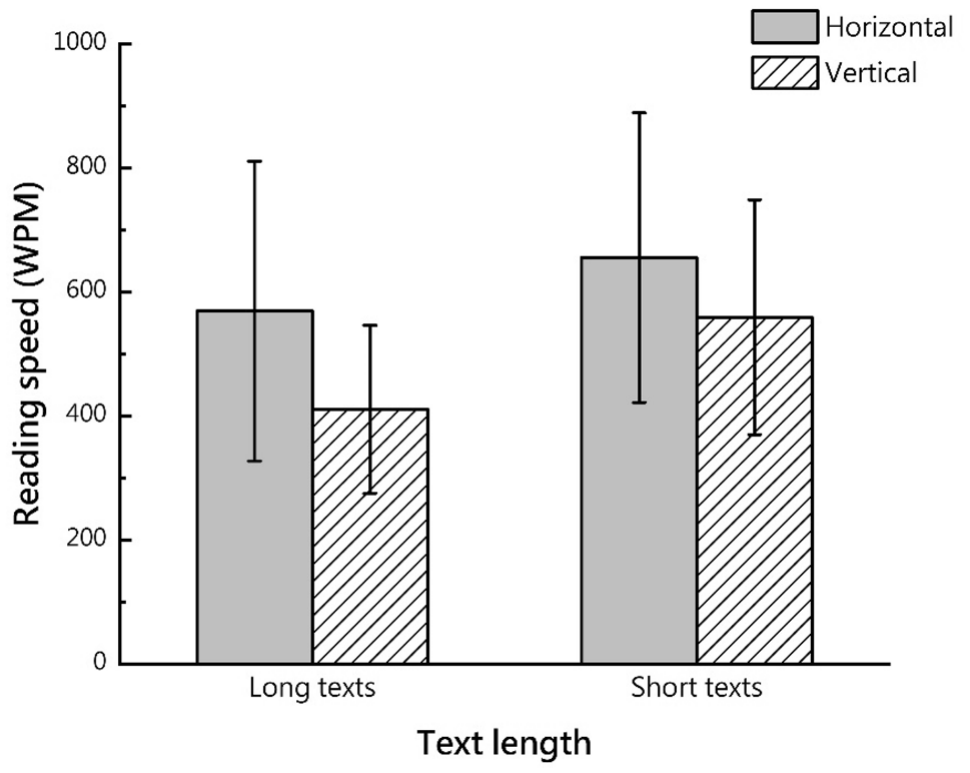
Mean and standard deviation of reading speed.

The descriptive statistics and RM-ANOVA results for reading speed are presented in Fig. 1 and Table 1, respectively.

**Table 1:** Participants’ reading speed.

	Long text		Short text	
	Horizontal	Vertical	Horizontal	Vertical
Mean	569.20	410.85	655.44	559.25
SD	241.752	135.396	233.635	189.396
Text length (P)	** < 0.001**			
Text direction (P)	**0.006**			
Text length*text direction (P)	0.154			

Significant values are in bold.

The interaction effect between text length and direction was not significant. The main effect of text size was significant (F (1, 66) = 29.590, p < 0.05; effect size partial η^2^ = 0.310), and the reading speed of the long text was higher than that of the short text. The main effect of text direction was significant (F (1, 66) = 8.122, p = 0.006; effect size partial η^2^ = 0.802), and participants read faster with horizontal text than vertical text.

### Memory

#### Number of correct answers

**Figure 2:**
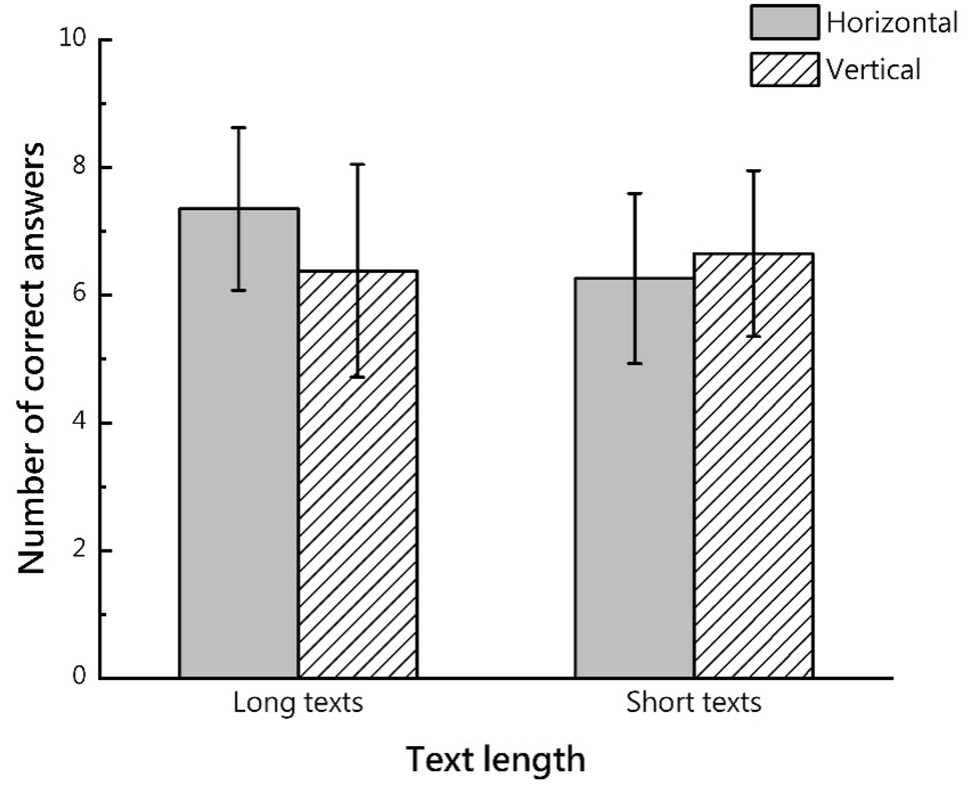
Mean and standard deviation of number of correct answers.

The results of the descriptive statistics for the number of correct answers and the RM-ANOVA results are presented in Fig. 2 and Table 2, respectively.

**Table 2:** Number of participants’ correct answers.

	Long text		Short text	
	Horizontal	Vertical	Horizontal	Vertical
Mean	7.35	6.38	6.26	6.65
SD	1.276	1.670	1.333	1.300
Text length (P)	**0.040**			
Text direction (P)	0.294			
Text length*text direction (P)	**0.001**			

Significant values are in bold.

The main effect of text length was significant (F (1, 66) = 4.390, p = 0.040; effect size partial η^2^ = 0.062). An interaction effect between text length and direction was also observed (F (1, 66) = 11.847, p = 0.001; effect size partial η^2^ = 0.152). Regarding text length, a simple effects analysis showed that the number of correct answers for the horizontal text was greater than that for the vertical text (F (1, 66) = 7.250, p = 0.009; effect size partial η^2^ = 0.099) in the case of the long text, whereas text direction had no significant effect on the number of correct answers for the short text. Regarding text direction, the number of correct answers for the horizontal long text was greater than that for the short text (F (1, 58) = 13.140, p < 0.05; effect size partial η^2^ = 0.188), whereas the effect of text length on the number of correct answers was not significant for vertical text.

#### Reaction time

**Figure 3:**
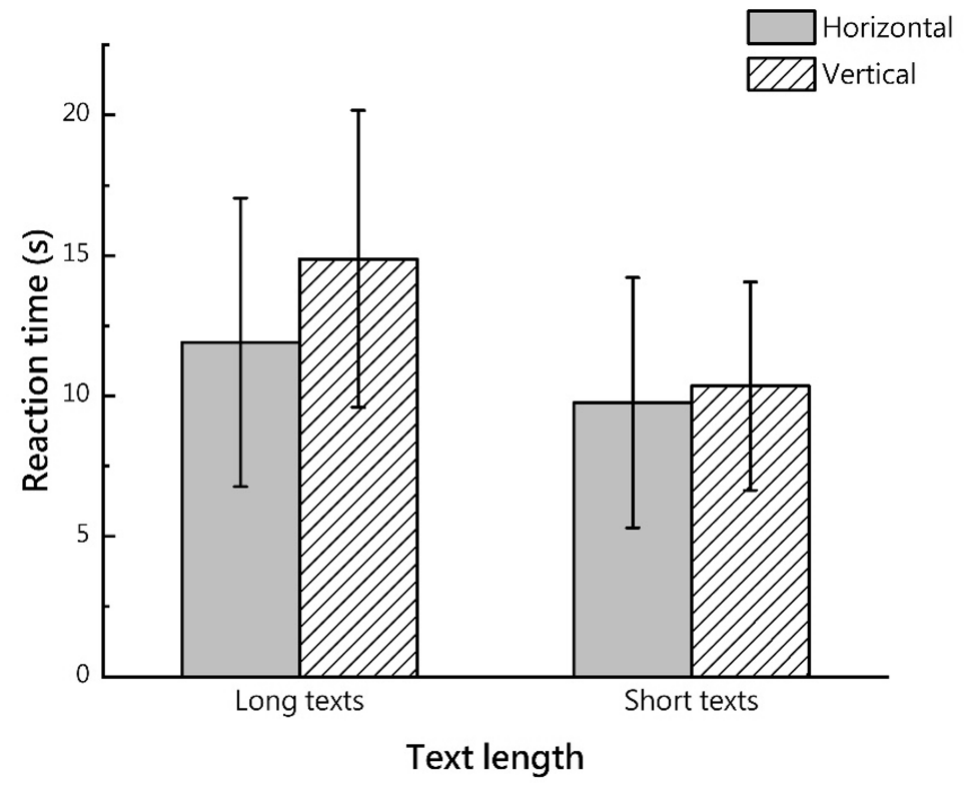
Mean and standard deviation of reaction time.

The results of the descriptive statistics for reaction time and those of the RM-ANOVA are presented in Fig. 3 and Table 3, respectively.

**Table 3:** Participants’ reaction time.

	Long text		Short text	
	Horizontal	Vertical	Horizontal	Vertical
Mean	11.91	14.88	9.76	10.35
SD	5.138	5.285	4.451	3.707
Text length (p)	< **0.001**			
Text direction (p)	0.095			
Text length*text direction (p)	**0.008**			

Significant values are in bold.

There was a significant interaction between text length and direction (F (1, 66) = 7.480, p = 0.008; effect size partial η^2^ = 0.102). Regarding text length, a simple effect analysis showed that the reaction time for vertical text was longer than that for the horizontal text (F (1, 66) = 5.515, p = 0.022; effect size partial η^2^ = 0.077) in the case of the long text, whereas there was no significant effect of text direction on reaction time in the vertical text. Whether horizontal (F (1, 66) = 12.177, p = 0.001; effect size partial η^2^ = 0.156) or vertical (F (1, 66) = 54.129, p < 0.001; effect size partial η^2^ = 0.451), the reaction time for the long text was longer than that for the short text.

### Task load

**Figure 4:**
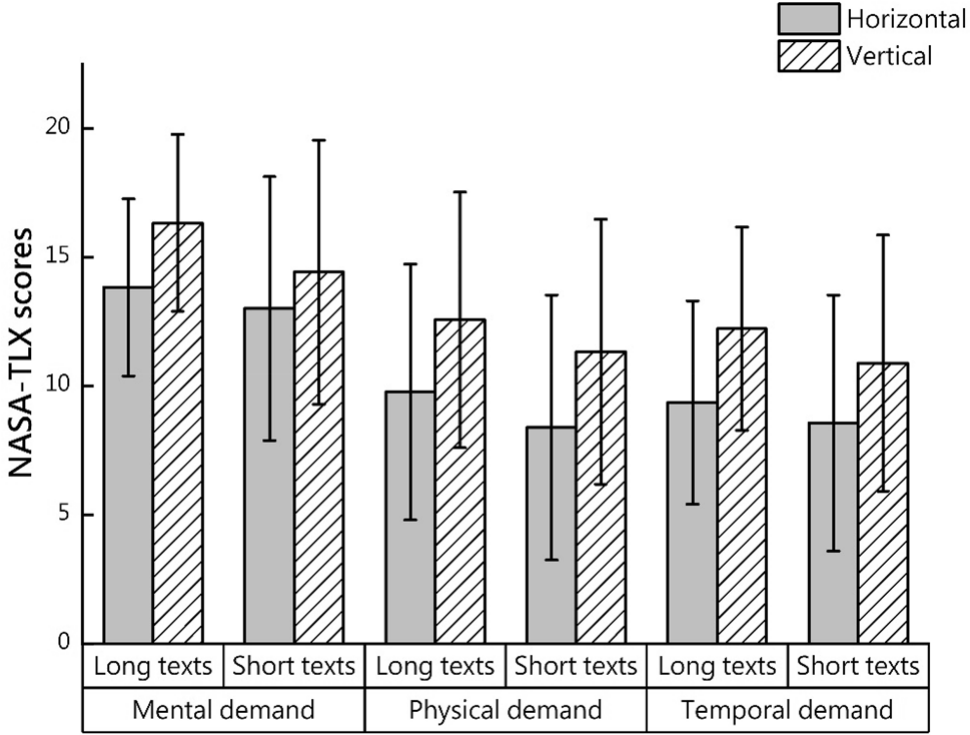
Mean and standard deviation of NASA-TLX scores.

The NASA-TLX scale was used to assess the participants’ task load, and the results of the descriptive statistics and analysis of variance are displayed in Fig. 4 and Table 4, respectively. Overall, no significant interaction effect was observed between text length and text direction.

**Table 4:** Participants’ NASA-TLX scores.

Dimension		Long text		Short text	
		Horizontal	Vertical	Horizontal	Vertical
Mental demand	Mean	13.82	16.32	13.00	14.41
	SD	3.688	3.435	3.482	5.123
	Text length (P)	**0.003**			
	Text direction (P)	**0.027**			
	Text length*Text direction (P)	0.221			
Physical demand	Mean	9.76	12.56	8.38	11.32
	SD	4.973	4.962	4.236	5.139
	Text length (P)	**0.030**			
	Text direction (P)	**0.006**			
	Text length*text direction (P)	0.901			
Temporal demand	Mean	9.35	12.21	8.56	10.88
	SD	4.396	3.945	4.032	4.971
	Text length (P)	**0.043**			
	Text direction (P)	**0.007**			
	Text length*text direction (P)	0.899			
Performance	Mean	9.79	8.68	9.74	10.53
	SD	4.154	3.616	3.934	4.223
	Text length (P)	0.123			
	Text direction (P)	0.836			
	Text length*text direction (P)	0.101			
Effort	Mean	13.74	14.71	13.29	13.62
	SD	3.646	3.623	3.344	4.818
	Text length (P)	0.126			
	Text direction (P)	0.425			
	Text length*text direction (P)	0.514			
Frustration	Mean	11.79	12.97	11.15	11.29
	SD	4.772	5.828	4.201	5.750
	Text length (P)	0.086			
	Text direction (P)	0.537			
	Text length*text direction (P)	0.443			

Significant values are in bold.

Regarding mental demand, the main effect of text direction was significant (F (1, 66) = 9.630, p = 0.003; effect size partial η^2^ = 0.127), and the mental demand for the vertical text was higher than that for the horizontal text. The main effect of text length was significant (F (1, 66) = 5.144, p = 0.027; effect size partial η^2^ = 0.072), and the mental demand for the long text was higher than that for the short text.

Regarding physical demand, a main effect of text direction was found (F (1, 66) = 4.940, p = 0.030; effect size partial η^2^ = 0.70), and the physical demand for vertical text was higher than that for horizontal text. The main effect of text length was significant (F (1, 66) = 7.975, p = 0.006; effect size partial η^2^ = 0.108), and the physical demand for the long text was higher than that for the short text.

Regarding temporal demand, there was a main effect of text direction (F (1, 66) = 4.253, p = 0.043; effect size partial η^2^ = 0.061), and the temporal demand for the vertical text was higher than that for the horizontal text. The main effect of text length was significant (F (1, 66) = 7.863, p = 0.007; effect size partial η^2^ = 0.106), and the temporal demand for the long text was higher than that for the short text.

## Discussion

This study investigated the effects of text direction on reading performance when reading long or short texts and assessed reading speed, memory, and task load by recording reading time, performing an R/K test, and measuring task load using the NASA-TLX scale after each reading. The results partially confirmed H1, H2, and H3 as follows:

(i) Horizontal reading speed was faster than vertical reading speed regardless of text length.
(ii) Horizontal reading had a better memory effect than vertical reading when reading long text, whereas the effect of text direction on memory was not significant for short text.
(iii) Long texts were reacted significantly slower than short texts.
(iv) Horizontal reading imposed fewer mental, physical, and temporal demands than vertical reading, regardless of text length.

The reading speed results showed that participants read horizontal text significantly faster than vertical text for both long and short texts. This is because in the observer’s binocular visual field, the vertical range is smaller than the horizontal range^34^, horizontal eye movements require less effort than vertical eye movements^35^, and horizonal eye movements decrease response time^38^. This supports the findings of Sun, Morita^29^, Chan and Ng^4^ and Wang, Cui^14^, who reported that Chinese adults read horizontally faster than vertically. Previous studies have shown that the visual span of vertical Chinese reading is approximately half that of horizontal Chinese reading^29^; however, present study differs from those of Miles and Shen^27^ and Chen and Carr^28^. This can be explained by the differences in participants' horizontal and vertical textual reading skills^29^, because in the early stages, readers have little experience in horizontal reading. The participants in this study were Chinese university students who were exposed to both horizontal and vertical texts in their daily lives and vertical classical books in their extra readings.

Regarding memory, this study showed that when reading long texts, participants had more correct answers and shorter reaction times for horizontal text than for vertical text, suggesting that participants had better memory for long horizontal texts. However, the effect of text direction on the number of correct answers and reaction time was not significant when reading short texts, and in the case of horizontal text, memory did not display a substantial advantage. The results for long texts confirm that cognition is better when reading horizontal rather than vertical text^14,33^. This may be attributed to the fact that horizontal eye movements increase interhemispheric brain activity^36,37^ and recognition sensitivity^38^. However, for short texts, the result may be partially explained by the fact that vertical reading speed is slower and browsing time is longer than in horizontal reading^29^; thus, cognitive enhancement can be significant only when the text is long enough^17^. In addition, long texts were reacted significantly slower than short texts, which was consistent with previous research that in a single highlighting task, the performance with a long text was not effective as that with a short text. This is probably because longer texts increase the need for a working memory system, whereas shorter ones are more dependent on decoding capacity^17,41^.

Regarding task load, both text direction and length significantly impacted mental, physical, and temporal demands. Vertical and long texts had higher scale scores; that is, the task load of the participants was higher when reading vertical or long texts. This may be because vertical eye movements require more effort than horizontal eye movements, increasing the difficulty of searching for and processing information^30,31^. This result supports the findings of Chan and Ng^4^ that horizontal text was significantly better than vertical text in terms of subjective preferences based on comfort, ease, and fatigue. Moreover, long text often results in low sentiment scores^16,39^. This may explain why task load scores were higher for long texts.

## Conclusion

Given the high flexibility of Chinese typesetting in the horizontal and vertical directions, this study explored the effects of text direction and length on reading performance by measuring participants’ reading speed, memory, and task load while reading texts of different directions and lengths from the perspectives of reading performance and cognitive efficiency. Our findings have significant implications for understanding how Chinese text arrangement affects reading performance. These results add to the rapidly expanding field of Chinese typography and further study the effect of text direction on memory and cognitive load. Designers should consider text length and choose the right text direction to improve users’ reading experience, memory levels, and learning efficiency, as well as to reduce task load. Moreover, this study may be particularly valuable for the design of advertisements, books, newspapers, and textbooks, providing suggestions and guidelines for designers and educators. The shapes of Chinese characters make them flexible in both horizontal and vertical writing, and the choice of writing direction is affected by numerous factors. The current popularity of horizontal writing does not mean that Chinese text itself is not suitable for vertical writing and reading.

Despite its many contributions, this study had several limitations. First, all participants were college students whose reading experiences and abilities may differ from those of other age groups and whose familiarity with different text directions may have an impact on the results. Second, this experiment included only two lengths of scientific and technological articles as reading materials; thus, it is necessary to further explore the impact of other types and lengths of articles on reading. Third, the memory tests were all presented horizontally for horizontal or vertical stimuli, which could be a factor in recall performance, while the results in Table 2 show no main effect of text direction on the number of correct answers. Future studies should further examine these effects for greater clarity. Finally, future research should include physiological indicators (e.g., eye movements and electroencephalogram) to provide stronger evidence of cognitive mechanisms and reading performance.

## Data Availability

The data and materials supporting the findings of this study are available from the corresponding author upon reasonable request.
