# Author Correction: The effects of text direction of different text lengths on Chinese reading

**DOI:** 10.1038/s41598-023-37597-w

**Published:** 2023-06-28

**Authors:** Yanqun Huang, Yifan Dong, Zhaojun Jiang, Peng Zhang, Jutao Li, Junyu Yang

**Affiliations:** 1 Key Laboratory of Mechanism Theory and Equipment Design of Ministry of Education, Tianjin University, Tianjin, 300354 China; 2 Tianjin Ren’ai College, Tianjin, 301636 China; 3 School of Automobile NCO, University of Army Military Transportation, Bengbu, 233011 Anhui China

Correction to: *Scientific Reports* 10.1038/s41598-023-35859-1, published online 29 May 2023

The original version of this Article contained an error in the Introduction section, where

“For thousands of years, the traditional Chinese writing layout has followed a vertical arrangement (i.e., the word order is from top to bottom, and the line order is from left to right), reflecting long-standing Chinese culture and expressing an Oriental manner of cognition^8^.”

now reads:

“For thousands of years, the traditional Chinese writing layout has followed a vertical arrangement (i.e., the word order is from top to bottom, and the line order is from right to left), reflecting long-standing Chinese culture and expressing an Oriental manner of cognition^8^.”

The original Article has been corrected.

